# Auditing Referrals Outcome From the Inverness General Practice to the Adult Community Mental Health Team

**DOI:** 10.1192/bjo.2026.11818

**Published:** 2026-06-30

**Authors:** Patience Otaniyen, Amy Macaskill, Eve Fordyce

**Affiliations:** NHS Highland, Inverness, United Kingdom

## Abstract

**Aims:**

1. To examine the referrals from the Inverness General Practice (GP) to the Community Mental Health Team (CMHT) in line with available referral guidelines and to ascertain reasons for referral rejections.

2. To propose and implement recommendations towards improving outcomes.

**Methods:**

Two cycles of clinical audit was completed for patients referred from the GP to the CMHT in January - June 2024 (first cycle) and May - October 2025 (second cycle). Electronic records of all 1048 patients referred during both audit cycles were reviewed and examined against the NHS Highland CMHT referral guidelines. Reasons for referral rejections were further explored. These Outcomes were discussed at the Primary Care/Secondary care Mental Health Interface Meetings and the NHS Highland CMHT referral guidelines were updated. Following this, the second cycle of the audit was completed.

**Results:**

During the first cycle, the CMHT received 421 referrals, out of which 214 (51%) were accepted and assessed while 194 (46%) were rejected largely for reasons of insufficient information,symptoms severity not being sufficient for secondary care input and no clear role for the CMHT.

Six (6) months after the CMHT referral guidelines was updated and GPs were encouraged to use it as a basis for referral to the CMHT, the second audit cycle was completed, with the CMHT receiving 627 referrals during this period, with 439 (70%) accepted while 188 (30%) were rejected.

This reflects about 19% improvement in the number of referrals meeting referral criteria and being accepted for further assessment by the CMHT and 16% decrease in number of rejected referrals, when compared with the first audit cycle.

**Conclusion:**

Undesirable referral outcomes, like referral rejections, are a significant concern within the NHS and can lead to delays in patient care, administrative inefficiencies and compromised health outcomes. It can also negatively impact the working relationships between healthcare providers involved in sending and managing referrals.

It is therefore important to understand the reasons behind referral rejections and to reflect on how to address this to ensure that patients are able to access professional care when needed. Understanding these challenges within the NHS context is crucial to devising effective solutions to mitigate referral rejections.

